# The Bromodomain and Extra-Terminal Domain (BET) Family: Functional Anatomy of BET Paralogous Proteins

**DOI:** 10.3390/ijms17111849

**Published:** 2016-11-07

**Authors:** Yasushi Taniguchi

**Affiliations:** Division of Basic Molecular Science and Molecular Medicine, School of Medicine, Tokai University, Isehara, Kanagawa 259-1193, Japan; ytanigu@is.icc.u-tokai.ac.jp; Tel.: +81-0463-93-1121; Fax: +81-0463-96-2892

**Keywords:** Bromodomain and Extra-Terminal Domain (BET), bromodomain, histone acetylation, gene transcription, BET inhibitor

## Abstract

The Bromodomain and Extra-Terminal Domain (BET) family of proteins is characterized by the presence of two tandem bromodomains and an extra-terminal domain. The mammalian BET family of proteins comprises BRD2, BRD3, BRD4, and BRDT, which are encoded by paralogous genes that may have been generated by repeated duplication of an ancestral gene during evolution. Bromodomains that can specifically bind acetylated lysine residues in histones serve as chromatin-targeting modules that decipher the histone acetylation code. BET proteins play a crucial role in regulating gene transcription through epigenetic interactions between bromodomains and acetylated histones during cellular proliferation and differentiation processes. On the other hand, BET proteins have been reported to mediate latent viral infection in host cells and be involved in oncogenesis. Human BRD4 is involved in multiple processes of the DNA virus life cycle, including viral replication, genome maintenance, and gene transcription through interaction with viral proteins. Aberrant BRD4 expression contributes to carcinogenesis by mediating hyperacetylation of the chromatin containing the cell proliferation-promoting genes. BET bromodomain blockade using small-molecule inhibitors gives rise to selective repression of the transcriptional network driven by c-MYC These inhibitors are expected to be potential therapeutic drugs for a wide range of cancers. This review presents an overview of the basic roles of BET proteins and highlights the pathological functions of BET and the recent developments in cancer therapy targeting BET proteins in animal models.

## 1. Introduction

In *Drosophila*, the maternal effects gene, *fsh*, plays critical roles in establishing segments and specifying their identities during embryonic development [1]. The *RING3* gene, found in the class II region of the human major histocompatibility complex (MHC) has substantial homology with the *fsh* gene [2]. The yeast *Bdf1* gene encodes a transcription factor that is required for sporulation [3,4]. A comparison of the Bdf1 protein with the FSH and RING3 proteins reveals three regions of amino acid sequence similarities including two tandem bromodomains and an extra-terminal domain [3,5]. The syntenic chromosomal areas in vertebrates are believed to have been generated by the repeated duplication of ancestral genes [6]. In human, the *ORFX* [7], *MCAP* [8], and *BRDT* [9] genes have a paralogous relationship with the *RING3* located in the MHC region [10,11]. The proteins produced from these genes also possess two tandem bromodomains and an extra-terminal domain. The group of proteins containing these three domains is termed the Bromodomain and Extra-Terminal Domain (BET) family. Based on the structural and functional similarities among the four paralogous genes, mammalian *Ring3*, *Orfx*, *Mcap*, and *Brdt* are simply named as *Brd2*, *Brd3*, *Brd4*, and *Brdt*, respectively.

In *Drosophila*, the *brahma* (*brm*) gene encodes a bromodomain-containing protein that is required for transcriptional activation of several *Hox* genes [12]. Tetrahymena histone acetyltransferase (HAT) A is a homolog of the yeast Gcn5p that is a bromodomain-containing transcriptional activator [13]. Further, biochemical analysis has identified Gcn5p as a HAT catalytic subunit, suggesting that histone acetylation is linked to the transcriptional activation of genes [13]. Most of the HAT-associated transcriptional activators contain bromodomains [14,15], which can interact specifically with acetylated lysine [16]. The bromodomain that is functionally linked to the HAT activity of transcriptional activators serves as a chromatin-targeting module deciphering the histone acetylation code [17,18]. In mouse cell lines, BRD4 plays crucial roles in controlling cell growth by regulating the expression of transcription factors [8]. This regulation requires recognition of the histone acetylation code by the Brd4 bromodomains [19]. BRD2 selectively interacts with acetylated lysine 12 on histone H4, indicating the specificity of histone recognition by the bromodomains [20]. BRD4 is bound to the positive transcription elongation factor b (P-TEFb) that is involved in most RNA polymerase II (RNA Pol II)-dependent transcription processes, and positively regulates P-TEFb activity in transcription [21,22]. BRD2 and BRD3 specifically recognize the histone acetylation code and allow RNA Pol II to transcribe through nucleosomes in an in vitro transcription system [23]. To facilitate transcription, Brd4 functions on distal enhancers as well as on gene bodies by interacting with the acetylated histones through bromodomains [24,25]. These facts provide evidence that the BET proteins regulate gene transcription through epigenetic interactions between bromodomains and acetylated histones.

A genome wide approach for the characterization of nucleosomes decoded by the double bromodomain BET factors shows that direct binding of BRD4 to acetylated nucleosomes associated with transcribed genes is required for their proper expression [26]. Global transcriptome analysis identifies BRDT as a transcriptional regulator that controls the expression of over 3000 genes responsible for the progression of meiosis during spermatogenesis [27]. On the other hand, the aberrant expression of BET promotes oncogenesis, blocking cell differentiation and driving the growth of cells. NUT midline carcinoma (NMC) is caused by a translocation-derived fusion protein, BRD4-NUT or BRD3-NUT, which hyperacetylates the nucleosomal domains including the anti-differentiation genes [28,29]. These studies have demonstrated that the BET proteins, which are epigenetic regulators of gene transcription, are strongly implicated in the regulation of cell growth and differentiation. In mouse models of NMC, JQ1, a small molecular BET inhibitor that binds specifically to bromodomains, promotes the differentiation and regression of tumor cells and contributes to prolonged survival [30]. In addition, a growing body of recent work highlights the pre-clinical efficacy of BET inhibitors including JQ1 in a wide range of malignancies [31,32,33]. BET inhibitors are used not only to validate their therapeutic potential in many cancers, but also to demonstrate that BET mediates the transcriptional regulation underlying learning and memory in mice [34].

Since FSH was first found to be a morphogenetic regulator in *Drosophila*, the roles of mammalian BET proteins, FSH counterparts, have been conclusively determined by a well-defined analysis. On an earlier occasion, interest in the functional analysis of BET was directed toward the elucidation of its basic roles as a transcriptional regulator. However, recent identification of the carcinogenic activity induced by aberrant BET expression and small molecular BET inhibitors has attracted the interest of many researchers in cancer therapy. This review article presents an overview of the basic roles of BET proteins in transcriptional regulation, and highlights the pathological roles of BET in oncogenesis and latent viral infection.

## 2. Genomic Organization of Bromodomain and Extra-Terminal Domain (BET) Family Genes and Structure of the Proteins Encoded by These Genes

**Figure 1:**
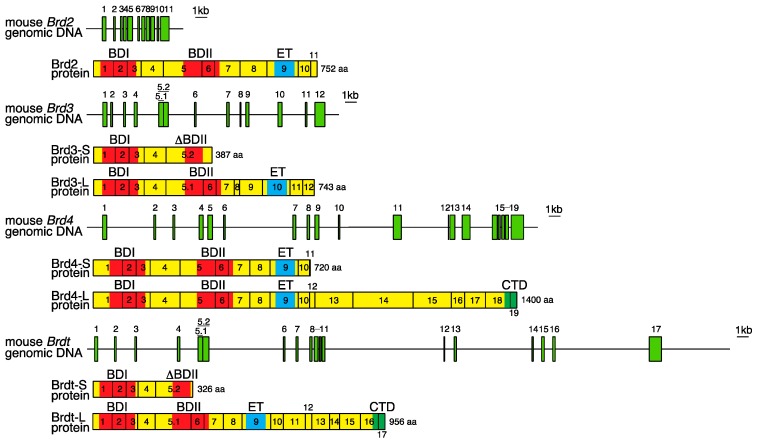

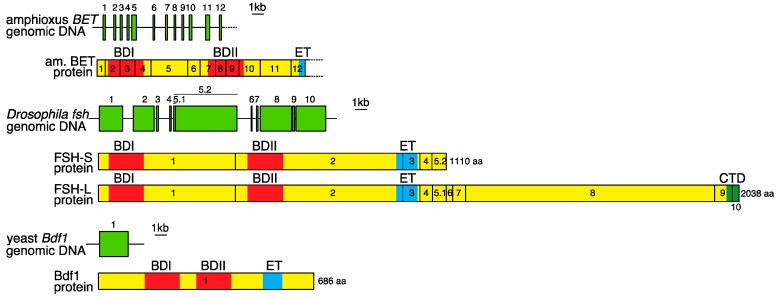
Structure of the mouse *Bromodomain and Extra-Terminal Domain* (*BET*) (*Brd2*, *Brd3*, *Brd4*, and *Brdt*), amphioxus *BET*, *Drosophila* *fsh* and yeast *Bdf1* genes, and the proteins encoded by these genes. Rectangles filled in yellowish green represent individual exons on genomic DNA. Long and short forms of an exon, which share the same nucleotide sequence, are classified by a decimal. Successive rectangles filled in yellow represent the primary structure of the proteins. The numbers in rectangles show areas encoded by the corresponding exons. Red, blue, and green areas indicate bromodomains (BDI, BDII, and truncated **∆**BDII), extra-terminal domains (ET), and C-terminal domains (CTD), respectively. The total number of amino acids comprising the protein is represented on the right of each primary protein structure. In amphioxus, partial structures are depicted on the basis of the information in the Data Bank. The nucleotide sequences of *BET* genes and amino acid sequences of BET proteins are based on the database information provided in the following accession numbers: Brd2: D89801 and AB212273; Brd3-S: AB212272; Brd3-L: AB206708; Brd4-S: AF461396; Brd4-L: AF273217; Brdt-S: AB208640; Brdt-L: AF358660; amphioxus BET: AF391288; FSH-S: M23222; FSH-L: M23221; and Bdf1, Z18944.

**Figure 2:**
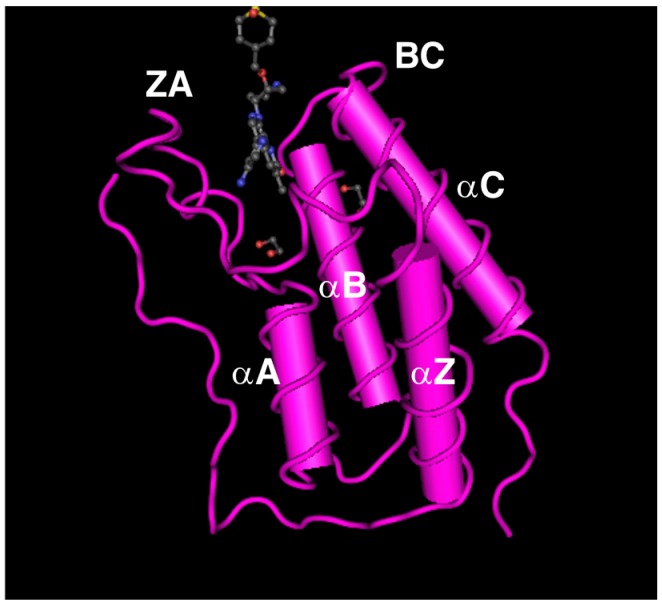
The tertiary structure of the human BRD4-BDI complex with the inhibitor JQ1. A long loop (loop ZA) connects helices αZ and αA, and another loop (loop BC) connects helices αB and αC. A pocket-shaped region formed by these two loops is a binding site for JQ1 or an acetylated lysine residue in histones. The small molecule seen in the pocket is the inhibitor JQ1. The structure of BRD4-BDI is based on the NCBI database information provided in the accession number 3MXF_A.

Paralogous genome regions in vertebrates, including the *BET* family genes, are presumed to have arisen by several rounds of genome-wide duplication [10,11]. In mammals, four BET paralogous proteins (BRD2, BRD3, BRD4, and BRDT) have been reported to exhibit similar amino acid sequences, domain organization, and some functional properties. The domain organization of mammalian BET proteins is conserved in orthologues including *Drosophila* FSH and *Saccharomyces cerevisiae* Bdf1 and Bdf2. The exon-intron organization of mammalian *BET* genes and the primary structure of BET proteins are shown in Figure 1, in comparison with those of amphioxus, *Drosophila*, and yeast. The coding region of the *Brd2* gene consists of 11 exons, spanning more than 6 kb of genomic DNA [2,35,36]. The coding region of *Brd3* consists of 12 exons spread over more than 20 kb of genomic DNA [7]. Nineteen coding exons of *Brd4* and 17 coding exons of *Brdt* span more than 39 and 52 kb of genomic DNA, respectively [8,9]. The BET family proteins essentially contain two tandem bromodomains (BDI and BDII) and an extra-terminal (ET) domain. The bromodomain is a conserved sequence of ~110 amino acids that structurally forms 4 α-helices (αZ, αA, αB, and αC) and 2 loops (ZA and BC), and can bind to acetyl-lysine residues in histones and other proteins (Figure 2) [37,38]. The bromodomain is required for the epigenetic regulation of gene transcription by BET proteins, through interaction with nucleosomes within chromatin [21]. The ET domain is a conserved region of ~80 amino acids that fulfills its regulatory function by recruiting specific effector proteins [39]. As shown in Figure 1, short variants of Brd3 and Brdt (Brd3-S and Brdt-S), generated by using a longer form of exon 5 as a terminal exon during the process of alternative splicing, show truncated BDII and lack ET. These short variants may be competitors of long isoforms (Brd3-L and Brdt-L) for acetyl-lysine motifs; however, their functions remain to be elucidated. Long variants of Brd4, Brdt, and *Drosophila* FSH (Brd4-L, Brdt-L, and FSH-L) possess the C-terminal domain (CTD) at their carboxy terminal region that is a conserved region of ~40 amino acids. The CTD interacts with the positive transcription elongation factor b (P-TEFb), which phosphorylates serine residues of the RNA Pol II C-terminal motif (CTM) [40]. Further, the CTD is identified to mediate P-TEFb activation from the inactive ribonucleoprotein complex [41,42]. Brd2, Brd3, and Brd4 are ubiquitously expressed in various tissues of adult mice whereas Brdt is specifically expressed in the testis [43,44].

Amphioxus or lancelet belongs to the subphylum Cephalochordata and is regarded as similar to the archetypal vertebrate form. An investigation of the MHC paralogous regions revealed a single genomic region in amphioxus that corresponds to the mammalian MHC [11]. Further, analysis of the genomic region in amphioxus identified a gene equivalent to the mammalian *BET* genes. The protein encoded by this gene possesses two bromodomains and an extra-terminal domain (Figure 1). The exon organization of the amphioxus *BET* gene bears some resemblance to those of the mouse *BET* genes. The amphioxus and mouse *BET* genes are composed of more than 10 exons and encode the bromodomain I across three exons (Figure 1). Phylogenetic analysis of genes in these MHC paralogous regions shows that several rounds of genome-wide duplications occurred after the divergence of cephalochordates and vertebrates but before the jawed vertebrate (Gnathostomata) radiation [11].

## 3. Basic Functions of BRD2 and BRD3

### 3.1. BRD2 Plays a Role in Cell Growth and Neuronal Cell Generation

Human BRD2 protein is known to be a novel nuclear Ser/Thr kinase whose activity is increased upon cellular proliferation and is remarkably elevated in peripheral blood lymphocytes collected from acute and chronic lymphoma patients [45]. Although kinase activity has not been observed in mouse BRD2, which has more than 90% amino acid sequence identity with human BRD2 [35], FSH-S, which is a *Drosophila* counterpart of BRD2, is reported to be a Ser/Thr kinase [46]. An assay using 3T3 fibroblast cells co-transfected with a BRD2 expression vector and luciferase reporter vectors harboring *cis*-acting E2F sites shows that BRD2 stimulates E2F reporter activity [47]. E2F protein is a transcription factor that promotes the synthesis of proteins required for the G1/S transition during the cell cycle. Further, in anti-BRD2 antibody affinity chromatography, E2F protein is co-purified with BRD2 from an extract derived from HeLa nuclei or HEK293 whole cells [47,48]. These results indicate that BRD2 is a positive regulator that promotes E2F-dependent cell cycle progression.

Mice lacking *Brd2*, which were generated by a commercially available embryonic stem (ES) cell line in which the gene-trap vector is inserted into the *Brd2* locus, exhibit embryonic lethality and abnormalities in the neural tube where the gene is highly expressed [49,50]. Embryonic lethality is likely to be due to impaired cellular proliferation and enhanced cell death [49]. Association analysis in human genetics using single nucleotide polymorphisms and microsatellite markers in the critical genomic region has revealed that *BRD2* is a major susceptibility locus for juvenile myoclonic epilepsy (JME), a group of neurological disorders characterized by epileptic seizures [51]. Heterozygous *Brd2*^+/−^ mice are viable and overtly normal, but have a decreased tonic-clonic seizure threshold compared to *Brd2*^+/+^ wild type mice. Further, anatomical analysis of the brain shows that the number of GABAergic neurons in the neocortex and the striatum of *Brd2*^+/−^ mice are decreased, compared to those in *Brd2*^+/+^ mice [52]. These findings indicate that an insufficiency of BRD2 protein is associated with a decrease in the number of neuronal cells required for critical brain structures.

### 3.2. BRD2 and BRD3 Specifically Recognize Acetylated Histones through Their Bromodomains to Promote Transcription of Genes Required for Determining Cell Identities

Fluorescence resonance energy transfer (FRET) is a measurable physical energy transfer phenomenon occurring between appropriate fluorophores in sufficient proximity. When a high-energy donor fluorescent molecule transfers energy to a low-energy acceptor fluorescent molecule, a photon of a specific wavelength emitted by the acceptor can be detected as the FRET signal [53]. FRET provides a useful approach to many biochemical studies on molecular interactions [54,55]. A flow cytometric adaptation of the FRET technique is employed to delineate general patterns of interactions between bromodomains and acetylated histones in living cells [20]. A FRET signal is observed in living HeLa cells transfected with both CFP-BRD2 and YFP-histone H4, but is not observed in cells transfected with both CFP-BRD2 and YFP-histone H1. Histones H3 and H2A produce little or no FRET signals. Furthermore, mutations in the lysines K5 and K12 of the N-terminal tail on H4 histone effectively abolish the FRET signals. Peptide precipitation of extracts from CFP-BRD2 transfected HeLa cells with H4 tail peptides, either unacetylated or acetylated at various lysines, shows that BRD2 binds strongly to H4 peptides acetylated at K12. Taken together, these findings provide valid evidence that BRD2 specifically recognizes the acetylated K12 of the N-terminal tail on histone H4.

**Figure 3:**
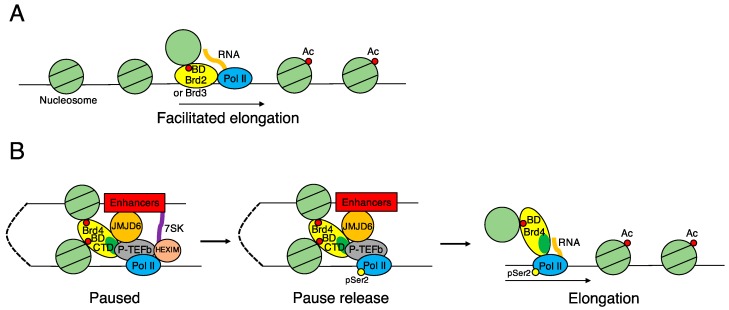
Transcriptional control by BET proteins. (**A**) Brd2 and Brd3 promote gene transcription. Interactions between their bromodomains (BD) and the acetylated lysine (Ac) in histones facilitate the passage of RNA Pol II to elongate nascent transcripts through hyperacetylated nucleosomes. The arrow indicates the direction of transcription; (**B**) Brd4 regulates gene transcription in the process of initiation and elongation. In the promoter-proximal region, RNA Pol II pauses due to inactivation of positive transcription elongation factor b (P-TEFb), forming a complex with the 7SK small nuclear RNA (snRNA) and the HEXIM1 protein. Enhanced recruitment of P-TEFb by Brd4 causes Ser2 phosphorylation in Pol II, leading to Pol II release from the pause in transcription elongation. The pause release is also supported by the interaction of P-TEFb with Brd4 and JMJD6 associated with distal enhancers. Brd4 interacts with acetylated lysine through its bromodomains (the red circle in Brd4), and P-TEFb interacts with the Brd4 CTD (the green area in Brd4). Further, Brd4 promotes nascent RNA synthesis along the gene on hyperacetylated nucleosomes via its bromodomains.

Chromatin immunoprecipitation (ChIP) of nuclear extracts from HEK293 cells expressing FLAG-tagged BRD2 or BRD3 shows that BRD2- and BRD3-bound chromatin is significantly enriched in several acetylation marks associated with transcribed genes, including H4K5, H4K12, and H3K14 acetylation, but contains little dimethylated H3K9, which is a characteristic of transcriptionally inactive heterochromatin [23]. An in vitro transcription assay, with highly purified RNA Pol II and nucleosomal templates that are assembled with either hypo- or hyperacetylated histones purified from HeLa cells, shows that both BRD2 and BRD3 assist transcription from hyperacetylated chromatin templates much more efficiently than from hypoacetylated chromatin templates [23]. These results demonstrate that BRD2 and BRD3 proteins possess nucleosome chaperone activities that allow RNA Pol II to elongate transcripts through hyperacetylated nucleosomes (Figure 3A).

To fully elucidate how BET proteins decipher the histone codes, which are combinations of histone modifications such as acetylation and methylation on nucleosomes, ChIP of the nuclear extract from HEK293 cells expressing FLAG-tagged BET proteins and quantitative mass spectrometry for immunoprecipitated core histones have been conducted. Consequently, it was confirmed that nucleosomes associated with BRD2, BRD3, and BRD4 are mostly enriched in at least three acetylations of H4K5, H4K8, H4K12, and H4K16, and H3K4 dimethylation or H3K4 trimethylation [26]. To further determine where BET proteins reside on the genome, the DNA isolated from BET-bound nucleosomes has been deeply sequenced. More than half the BET-binding sites are found on genes including promoters, and the remaining sites are found on intergenic regions. The majority of these genes are highly and moderately expressed, and a few genes are transcriptionally silent [26]. Moreover, a ranked list of genes that are ordered based on the BET binding score within the promoter region of all annotated genes shows that an unusually large number of the highest-ranking promoters are derived from *HOX* genes [26]. Thirty-nine *HOX* genes that are arranged in four clusters on the mammalian genome encode transcription factors that determine cell and tissue identities in the embryo during development and in the adult [56,57,58]. Because the *Drosophila* FSH, a counterpart of mammalian BET proteins, has been reported as crucial for the activation of the *Ultrabithorax* *HOX* gene [46,59], the regulatory mechanism of *HOX* expression by BET proteins might be conserved across species. Collectively, these findings support the idea that BET proteins fulfill key roles as “readers” of the histone code to induce transcriptional activation of various genes that are required for determining cell identities.

BRD3 has been reported to directly interact with acetylated lysine residues of the transcription factor GATA1, which regulates the expression of all erythroid and megakaryocyte-specific genes [60]. Pull-down of extracts from GATA1-null erythroblasts (G1E cells) expressing normal or mutant forms of HA-tagged BRD3 using acetylated GATA1 peptides shows that lack of BDI interferes with the binding of BRD3 to acetylated peptides, suggesting that recruitment of BRD3 to GATA1-associated chromatin requires BDI [61]. G1E cells transfected with a *GATA1*-estrogen receptor fusion gene, termed as G1E-ER cells, express GATA1 in the presence of exogenous estradiol [62]. ChIP-sequencing (ChIP-seq) analysis using antibodies against GATA1 and BET proteins in G1E-ER cells in the absence and presence of estradiol reveals that BRD2, BRD3, and BRD4 occupy GATA1-bound loci, including the β-globin locus *Hbb* [63]. An increase in BRD3 occupancy at GATA1 sites following GATA1 activation is most significant as compared to that of BRD2 and BRD4 occupancy, suggesting that BRD3 recruitment is predominantly affected by GATA1 whereas BRD2 and BRD4 recruitment is regulated by factors besides GATA1 [63]. However, BRD3 ablation by small hairpin RNA (shRNA)-mediated knockdown in G1E-ER cells has little effect on the expression of GATA1-target genes upon GATA1 induction, suggesting that BRD3 is not essential for erythroid differentiation. In contrast, BRD2 deficiency markedly reduces the transcript levels of GATA1-target genes. Since BRD3 knockdown exacerbates the consequences of BRD2-deficiency on GATA1-activated gene expression, BRD2 and BRD3 may act synergistically in GATA1-mediated erythroid gene activation [63].

## 4. Basic Functions of BRD4

### 4.1. BRD4 Is Required for Cellular Proliferation

BRD4 is a nuclear protein widely expressed in mammalian tissues, and its expression is induced by mitogen stimulation during the G_0_/G_1_ transition in lymphocytes, prior to entry into the S phase [8]. BRD4 localized in whole nuclei during the interphase becomes exclusively associated with chromosomes during the M phase when most of the nuclear regulatory factors are released into the cytoplasm in response to global stop of transcription [8]. Further, microinjection of an anti-BRD4 antibody into HeLa cell nuclei completely inhibits mitotic entry, suggesting that BRD4 plays a role in G_2_/M transition [8]. In a technique of fluorescence loss in photobleaching (FLIP), an area within living cells is repeatedly bleached, and the loss of fluorescence in areas that are distinct from the bleached area is monitored [64,65]. The rate of fluorescence signal loss is dependent on the mobility of a protein, and is decreased in slower mobile proteins. FLIP assays using P19 embryonic carcinoma cells transfected with GFP-tagged BRD4 show that GFP-BRD4 fluorescence is lost more slowly in the presence of trichostatin A (TSA), which is a histone deacetylase inhibitor, suggesting the affinity of BRD4 for chromatin is increased by histone acetylation [19]. Furthermore, mutations introduced into BRD4 bromodomains lead to more rapid loss of fluorescence signal in the presence of TSA, suggesting that bromodomains preferentially interact with acetylated histones [19]. These findings collectively indicate that BRD4 is associated with chromatin and chromosomes during the cell cycle, by interacting with acetylated histones via its bromodomains, and is necessary for cell cycle progression.

Using mouse embryonic stem (ES) cells transfected with a gene-trap vector for screening insertional mutations in developmentally regulated genes, a mouse mutant line in which an insertion generates a null allele of the *Brd4* gene was established [66]. *Brd4* heterozygotes display prenatal and postnatal growth defects associated with a reduced rate of cell growth. Embryos homozygous for a *Brd4* loss-of-function mutation die shortly after implantation because of their disability to form the inner cell mass (ICM) in blastocysts [66]. BRD4 knockdown by small interfering RNA (siRNA) microinjection into the blastomere of a two-celled mouse embryo leads to the reduction of ICM cells and inhibition of Nanog expression in these cells, suggesting that BRD4 regulates the expression of Nanog, which is required for maintaining the pluripotency of ES cells during early mammalian development [67]. These results provide evidence that BRD4 plays a critical role in cellular proliferation and ES cell self-renewal.

### 4.2. BRD4 Regulates Gene Transcription by Interacting with Acetylated Histones through Its Bromodomains

Immunoaffinity purification of nuclear extracts from HeLa cells expressing FLAG-tagged and HA-tagged BRD4 with an anti-FLAG or anti-HA antibody results in the precipitation of complexes composed of BRD4, Cdk9, and cyclinT1 [21,22]. This finding demonstrates that BRD4 interacts with Cdk9 and cyclinT1, which constitute the core positive transcription elongation factor b (P-TEFb). Active P-TEFb, which functions as a protein kinase, phosphorylates the RNA Pol II CTM, and results in releasing the Pol II from a pause in transcription elongation in the promoter-proximal region and promotes the efficient progression of Pol II along the gene [68,69,70]. About half of the cellular P-TEFb in HeLa cells also exists in an inactive complex with the 7SK small nuclear RNA (snRNA) and the HEXIM1 protein [71,72]. Immunoblotting of extracts from BRD4 overexpressing HeLa cells with an antibody specific to Ser2 or Ser5 CTM phosphorylation results in a specific increase in Ser2 CTM phosphorylation. Conversely, in HeLa cells that express BRD4-siRNA, Ser2 phosphorylation is substantially reduced. Moreover, BRD4 promotes the activity of a reporter gene driven by the HIV-1 long terminal repeat (LTR) promoter and cellular promoters such as *c-Myc* and *c-Jun* in a dose-dependent manner. These results indicate that BRD4 has a positive effect on Ser2 phosphorylation of the RNA Pol II CTM by enhancing the recruitment of P-TEFb [21]. Immunoprecipitation experiments using 293T cells transfected with FLAG-tagged BRD4 deletion mutants reveal that the C-terminal 34 amino acids of BRD4 are crucial for interaction with P-TEFb [40]. BRD4 has also been reported to be an atypical kinase that directly phosphorylates a Ser2 residue in the RNA Pol II CTM [73]. In vitro kinase activity assays have been performed using the following four peptides: recombinant P-TEFb, the CTM of RNA Pol II, a C-terminal segment of 54 amino acid residues in BRD4 that is termed as the P-TEFb interacting domain (PID), and HEXIM1, sequestering P-TEFb into an inactive complex. The result showed that an antagonistic effect of HEXIM1 on P-TEFb-mediated CTM phosphorylation is canceled in the presence of PID, indicating that BRD4 relieves the inhibition of P-TEFb by HEXIM1 rather than phosphorylating P-TEFb [42].

Mass spectrometry analysis of affinity-purified proteins from cell lysates of 293T cells stably expressing BRD4 Flag-HA-tagged fragments identified NSD3 and JMJD6 as interactors of the BRD4 ET domain [39]. NSD3 belongs to a subfamily of H3K36 methyltransferases [74], and JMJD6 is known to be a histone arginine demethylase [75]. Knockdown of NSD3 or JMJD6 using siRNA in C33A cells decreases the expression of BRD4 target genes by 2- to 3-fold, suggesting that NSD3 and JMJD6 are recruited to the regulated genes in a BRD4-dependent manner [39]. A pull-down assay of nuclear extracts from HEK293T cells with an anti-JMJD6 antibody results in the precipitation of BRD4 complexed with P-TEFb, suggesting that JMJD6 associates with the active form of the P-TEFb complex [24]. Whole genome ChIP-seq with an anti-Pol II antibody shows that knockdown of *JMJD6* or *BRD4* by siRNA in HEK293T cells significantly increases the Pol II occupancy rate at promoter-proximal regions and dramatically decreases its rate along the gene body, suggesting that association of JMJD6 and BRD4 promotes the Pol II promoter-proximal pause release [24]. Further, genome-wide ChIP-seq analysis with antibodies against several histone markers, JMJD6, and BRD4 identifies JMJD6- and BRD4-associated distal enhancers characterized by H3K4me1 [76], H3Ac, H4Ac, and H3K27Ac markers [24]. In concert, these findings raise the possibility that the next-nearest-neighbor interaction of the P-TEFb complex at the promoter-proximal region with BRD4 and JMJD6 co-bound to the distal enhancer permits the pause release for transcriptional elongation (Figure 3B).

In addition to the role of recruiting P-TEFb to release RNA Pol II from pausing in the promoter-proximal region, BRD4 has been reported to play a role in supporting the progression of Pol II through hyperacetylated nucleosomes by interacting with acetylated histones via its bromodomains [25,77]. Assays with NIH3T3 cells stimulated with interferon (IFN)-β show that IFN-induced gene transcription is markedly suppressed by JQ1, which inhibits BRD4-acetyl histone binding, or by a transfected short hairpin RNA (shRNA) plasmid causing BRD4 knockdown [77]. This result indicates that inducible gene transcription is dependent on interactions between BRD4 and the acetylated lysine residues in histones. Metagene analysis of chromatin-bound nascent RNA-seq reads of serum-responsive genes, activated by serum re-stimulation after starvation in 3T3 cells shows that JQ1 antagonizes the downstream process of transcriptional elongation rather than the typical pause-releasing event occurring in the proximal region of transcription start sites [25]. *c-Myc* and *Klf-4* are BRD4-dependent genes whose transcript levels are reduced upon BRD4 knockdown or JQ1 treatment. In BRD4-knockdown cells, wild type YFP-tagged BRD4 effectively rescues *c-Myc* and *Klf-4* expression, whereas YFP-BRD4 whose bromodomains are mutated does not rescue their expression. Analysis of nascent chromatin-bound RNA-seq reads across the *c-Myc* and *Klf-4* gene loci in NIH3T3 cells reveals that transcript elongation on these genes is clearly inhibited by JQ1. An in vitro transcription assay shows that BRD4 allows Pol II to transcribe through nucleosomes in a histone hyperacetylation dependent manner, and that JQ1 specifically inhibits the activity of BRD4 but not that of the histone chaperone FACT (facilitates chromatin transcription). In BRD4-knockdown cells, the short form of BRD4 lacking the C-terminal P-TEFb interacting domain (BRD4 short) rescues the expression of *c-Myc* and *Klf-4*, while the BRD4 short form whose bromodomains are mutated (BRD4 short mBD mutant) does not rescue the expression of these genes. ChIP and quantitative PCR (ChIP-qPCR) assays with an antibody specific for Ser2 CTM phosphorylation in RNA Pol II (Ser2P Pol II) reveal that Ser2P Pol II distribution on the *Klf-4* locus is shifted towards and beyond the transcription end site in BRD4-knockdown cells reconstituted with the short BRD4 compared with those reconstituted with the BRD4 short mBD mutant. These observations collectively support the idea that BRD4 facilitates Pol II progression along the gene body independently of P-TEFb as it traverses hyperacetylated nucleosomes by interacting with acetylated histones through its bromodomains [25]. This assistance of BRD4 for Pol II elongation is due to its histone-chaperone activity. Figure 3B illustrates the manner by which BRD4 contributes to gene transcription together with other components.

Rapid activation of immediate early genes (*IEGs*) in response to external signals is critical for the consolidation of synaptic modifications underlying synaptic plasticity and memory formation [78,79]. In cultured neurons isolated from mice, brain-derived neurotrophic factor (BDNF) causes a rapid increase in IEG transcripts. However, both JQ1-treatment and BRD4 knockdown by siRNA block the BDNF-induced increase in IEG expression [34]. These data support the idea that BRD4 recruits P-TEFb to promote Pol II phosphorylation and rapidly allows Pol II to elongate the IEG transcripts. Among the experimental models assessing cognitive functions in mice, the novel object recognition test can be evaluated by differences in the exploration time of novel and familiar objects [80]. If mice remember the previous objects, they will subsequently spend more time with a novel object. JQ1-injected mice showed no preference for novel objects compared with that of control mice, suggesting that JQ1 affects long-term memory [34]. Taken together, these findings indicate that BRD4 is critical for neuronal function and mediates the transcriptional regulation underlying learning and memory in mice. The heat shock response is a set of regulated responses to stress in the cell. The features of the heat shock response are the production of heat shock proteins and a partial inhibition of pre-mRNA splicing [81]. BRD4 has been identified as a regulator of the IFN-stimulated and oxidative stress-induced responses [77,82]. Alternative splicing analysis from RNA splicing data of heat shock-treated cells and heat shock-treated cells with BRD4 knockdown shows a significant increase in splicing inhibition, in particular intron retentions, in BRD4-depleted cells after heat shock, indicating that BRD4 prevents cells from heat stress-induced splicing inhibition [83]. Overall, BRD4 plays a critical role in controlling gene expression in response to external signals.

### 4.3. BRD4 Functions in Mitotic Bookmarking

Fluorescence recovery after photobleaching (FRAP) is a technique used to study protein mobility in living cells by measuring the rate of fluorescence recovery at the bleached site [84]. FRAP results are analyzed quantitatively to determine whether protein mobility is rapid or slow. FRAP analysis with a confocal laser-scanning microscope provides a crucial means to globally describe the binding dynamics of chromatin-associated proteins in living cells [85,86]. The recovery of the bleached fluorescence signal in wild-type chromatin-associated proteins is slower than in the binding-impaired mutants of chromatin-associated proteins, suggesting that an increase in binding affinity to chromatin gives rise to decreased recovery rate [85]. Fluorescently bleached GFP-BRD4 in NIH3T3 cells shows more rapid recovery in the G1 and G2 phases and metaphase/anaphase than that in the telophase during mitosis, indicating that BRD4 acquires increased binding affinity for chromatin at telophase when other nuclear factors begin to reassociate with chromatin and transcription restarts [87]. At telophase, the chromatin-binding BRD4 promotes P-TEFb-dependent phosphorylation of Ser2 in the CTM, which signifies an elongation state of Pol II. Subsequently, BRD4 has an impact on restarting the transcription of late M and early G1 genes, many of which are crucial for cellular functions in newly divided cells. These results suggest that BRD4 marks the M/G1 genes for transcriptional memory during mitosis and plays a role in the prompt initiation of their transcription in late mitotic and post-mitotic cells [87].

Transmission of transcriptional memory from mother to daughter cells is reported to be mediated by chromatin-based epigenetic bookmarks [20,88,89]. In the study of transcriptional activation kinetics, the doxycyclin (Dox)-inducible mammalian cells, U2OS-2-6-3, expressing pTet-on, mCherry-Pol II and MS2-YFP, allow real-time imaging of the dynamics of Pol II recruitment and mRNA synthesis during the cell cycle [89,90,91]. Both Pol II and MS2 signals induced by Dox in cells reach a plateau level much more rapidly during post-mitotic transcriptional reactivation than during interphase transcriptional activation, indicating that a gene bookmark is involved in post-mitotic transcriptional reactivation. The kinetic analysis of mRNA synthesis during post-mitotic transcriptional induction shows that signals of MS2 nascent transcripts reach a plateau level more slowly in BRD4 knockdown and BRD4 inhibitor-treated cells compared to those in control cells [89]. Collectively, these facts suggest that BRD4 acts as a gene bookmark for transcriptional reactivation in post-mitotic cells.

## 5. Basic Functions of BRDT

### 5.1. BRDT Is Essential for Spermatogenesis

*Brdt* is exclusively expressed in the testis [9,43,44]. Its expression restricted to the male germ line is initiated at the early spermatocyte stage during meiosis and persists throughout the post-meiotic stage during spermiogenesis [27]. Targeted mutagenesis has been carried out to introduce *Brdt**^∆^**^BDI^*, which lacks only the first of the two bromodomains, into the *Brdt* locus. *Brdt**^∆^**^BDI/**∆**BDI^* homozygous male mice are sterile and show impaired testicular histology with severely reduced sperm concentrations and abnormal sperm morphology, suggesting that *Brdt* is essential for normal spermatogenesis [92]. Furthermore, complete deletion of *Brdt* alleles results in meiotic arrest at the end of the prophase when the pairing chromosomes should undergo compaction in preparation for the first meiotic division [27]. Transcriptome analyses with wild type and *Brdt*^−/−^ pachytene spermatocytes show that BRDT governs the expression levels of more than 3000 genes, activating approximately two-thirds of these genes and repressing one-third of them [27]. *Ccna1* (*Cyclin A1*), one of the genes markedly activated by BRDT, is exclusively expressed in the male germ cell lineage, and is essential for spermatocytes to enter the first meiotic division [93,94]. Taken together, these findings indicate that the BRDT protein is a transcriptional regulator that controls the expression of genes responsible for meiotic progression during spermatogenesis.

### 5.2. BRDT Interacts with Various Proteins and Functions as a Transcriptional Regulator during Spermatogenesis

Immunoprecipitation of extracts from adult mouse testes with anti-Cdk 9 and anti-cyclin T1 antibodies showed BRDT as a factor binding to both Cdk 9 and cyclin T1, suggesting that BRDT as well as BRD4 is required for the recruitment of P-TEFb, which is known to bind the C-terminal region of BRD4 [27,40]. This finding is also supported by the fact that the C-terminal sequence of BRDT is strikingly similar to that of BRD4 (Figure 1). These data confirmed that BRDT is a true functional tissue-specific paralogue of BRD4. To determine the interacting partners of BRDT, immunoprecipitation of rat testicular nuclear extracts using an anti-BRDT antibody and mass spectrometry analysis of proteins derived from precipitates identified Smarce1 as an interacting partner of BRDT [95]. Smarce1 is a member of the SWI/SNF (SWItch/Sucrose Non-Fermentable) family and a component of the multimeric ATP-dependent chromatin remodeling complexes that regulate subunit interactions and transcriptional activation. A pull-down assay of a FLAG-tagged BRDT deletion mutant and recombinant Smarce1 mixture using an anti-Smarce1 antibody shows that N-terminal deletion interferes with co-immunoprecipitation of BRDT with Smarce1, suggesting that the N-terminal region is necessary for BRDT to associate with Smarce1 [95]. Comparative microarray analysis of RNA from wild-type and *Brdt**^∆^**^BDI/**∆**BDI^* mutant round spermatids reveals that, among the genes that are upregulated by BDI deletion, RNA splicing genes are enriched and that over 60% of these splicing genes have transcripts that lack the truncation of their 3′-untranslated region, that is typical of round spermatids [96]. Immunoprecipitation of mouse testicular lysates using an anti-BRDT antibody reveals the co-immunoprecipitation of Srsf2, Ddx5, Hnrnpk, and Tardbp that participate in RNA splicing, suggesting that BRDT complexes with these splicing proteins and functions as a part of the splicing machinery in the testis [96]. Further, BRDT has been reported to repress the expression of the testis-specific histone *H1t* during spermatogenesis by interacting with the histone deacetylase, HDAC1, the arginine-specific histone methyltransferase 5, PRMT5, and the Tripartite motif-containing 28 protein, TRIM28 [97]. Collectively, these results indicate that BRDT associates with a number of regulatory proteins such as P-TEFb and exerts its functions as a transcriptional activator or repressor during spermatogenesis.

### 5.3. BRDT Exerts a Function as a Chromatin-Remodeling Factor during Spermatogenesis by Interacting with Acetylated Histones

Chromatin remodeling assays with Cos7 cells transfected with *Brdt* cDNA show that ectopic expression of *Brdt* triggers dramatic reorganization of chromatin only after induction of histone hyperacetylation by TSA [98]. The same effect was confirmed by experiments in which nuclei isolated from TSA-treated mouse erythroleukemia (MEL) and 3T3 fibroblast cells are incubated with recombinant BRDT in vitro. Mutation in the first bromodomain compromises chromatin reorganization by insulating the BRDT protein from hyperacetylated histone H4 N-terminal tails [98]. In vitro remodeling assays with nuclei isolated from cultured rat haploid round spermatid cells show that exogenously added recombinant BRDT causes chromatin reorganization in the presence of histone deacetylase inhibitors such as sodium butyrate and TSA [95]. Isothermal titration calorimetry (ITC) is a quantitative technique that can determine the binding affinity of protein-protein interactions in solution [99]. An ITC analysis performed to study the binding of variously acetylated histone H4 peptides with recombinant BRDT (full length), BDI, and BDII, shows that a single bromodomain (BDI) of BRDT is responsible for the cooperative binding of two or more acetylation marks among K5, K8, K12, and K16 on the histone H4 tail [100]. Further, intramolecular FRET assays with a tandem fusion protein consisting of BRDT bromodomains and a histone H4 flanked by two different colored fluorescent proteins serving as the donor and acceptor fluorophores, show that COS7 cells expressing the fusion protein release a FRET signal only in the presence of TSA, suggesting that BRDT bromodomains interact with acetylated histones in living cells [101]. Overall, these findings indicate that BRDT is a key molecule that participates in chromatin remodeling by interacting with hyperacetylated nucleosomes via its bromodomains.

During the post-meiotic stages of spermatogenesis, haploid spermatids undergo extensive morphological changes including a striking chromatin reorganization and compaction [102]. During this process, the nuclear volume dramatically reduces, and genome-wide histone removal and their step-wise replacement by small basic proteins occur. Histones are first replaced by transition proteins (TPs), which are later followed by protamines. This exchange is associated with hyperacetylation of histone H4 [103]. Elongating spermatids from the testes of *Brdt**^∆^**^BDI/**∆**BDI^* mice contain as a large number of hyperacetylated histones as the normal elongating spermatids. However, immunohistochemical analysis of elongating spermatids from *Brdt**^∆^**^BDI/**∆**BDI^* mice shows that TPs and protamines synthesized in the cytoplasm do not enter the nuclei and that histone replacement does not occur [27]. This finding suggests that the first bromodomain, BDI, is required to reorganize chromatin architecture by mediating the replacement of acetylated histones at post-meiotic stages during spermatogenesis.

## 6. Pathological Functions of BET Proteins Leading to Disease

### 6.1. BRD4-NUT or BRD3-NUT Fusion Protein Causes NUT Midline Carcinoma

NUT midline carcinoma (NMC) is a rare but poorly differentiated and highly aggressive cancer of the squamous cell lineage that arises in midline structures [104,105]. NMC is cytogenetically characterized by a reciprocal translocation of the *NUT* (nuclear protein in testis) gene on the long arm of chromosome 15, with *BRD4* on chromosome 19p13.1 (t(15;19)(q14;p13.1)) or, in rare cases, with *BRD3* on chromosome 9q34.2 (t(15;9)(q14;q34.2)), leading to BRD4-NUT or BRD3-NUT fusion protein production by NMC cells [28,106]. Knockdown of BRD4-NUT or BRD3-NUT in NMC cell lines (TC797, PER-403, and 10326 cells) results in squamous differentiation and growth arrest [103]. Further, knockdown of BRD4-NUT in HCC2429 cells, an NMC cell line established from a t(15;19) *BRD4-NUT* translocation lung cancer patient [107], induces their transformation from spindle-shape mesenchymal morphology to squamous morphology, increased expression of cell differentiation markers, and a reduction in cellular proliferation rate [108]. These results suggest that BRD-NUT fusion proteins contribute to carcinogenesis by interfering with epithelial differentiation.

Immunohistochemical staining of Cos7 cells transfected with a BRD4-NUT expression vector and of HCC2429 cells using an anti-acetylated histone H4 antibody shows that the BRD4-NUT fusion protein forms speckled nuclear foci that contain hyperacetylated chromatin domains [108,109]. Immunoprecipitation experiments using extracts from Cos7 cells transfected with the HA-tagged NUT and HA-tagged BRD4-NUT expression vectors show that p300, a cellular histone acetyltransferase, is co-immunoprecipitated with an anti-HA antibody, suggesting that the NUT moiety of the fusion protein interacts with and recruits p300 [109]. This result is also supported by an immunohistochemical study showing the co-localization of BRD4-NUT with p300 in punctate nuclear foci that are observed in HCC2429 cells [109].

Treatment of HCC2429 cells with etoposide, an inducer of single- or double- strand DNA breaks, does not induce expression of the *p21* gene that is a direct target of the p53 transcription factor, whereas treatment of A549 lung cancer cells with etoposide causes *p21* expression. Knockdown of BRD4-NUT in HCC2429 cells induces efficient restoration of p21 expression, accompanied by accumulation of caspase 3, a member of the cascading reaction associated with apoptotic cell death, and E-cadherin, a marker of epithelial cell differentiation [109]. Moreover, treatment of the BRD4-NUT knockdown cells with a p300 inhibitor severely interferes with the induction of p53 target genes, suggesting that p300 activity is required to activate these genes after BRD4-NUT knockdown. Altogether, these findings demonstrate that sequestration of p300 into the BRD4-NUT foci principally drives oncogenesis leading to p53 inactivation, and that knockdown of BRD4-NUT releases p300 and regenerates p53-dependent regulatory mechanisms leading to cell differentiation and apoptosis.

Expression of BioTAP [110] -tagged BRD4-NUT causes the formation of hyperacetylated nuclear foci in 293T cells that are similar in number and appearance to those of endogenous BRD4-NUT in cultured NMC cells and in NMC tumor tissues [29]. ChIP-seq assays using chromatin obtained from 293T cells that express BioTAP-BRD4-NUT through tandem affinity purification of the BioTAP tag reveal more than 100 hyperacetylated chromatin domains reaching up to 2 Mb in size [29]. One of the long noncoding RNAs (lncRNAs), PVT1, is associated with prodigious domains that are commonly observed in all NMC cell lines and tissues examined. Treatment of NMC cells with siRNAs targeting PVT1 results in the expression of differentiation markers, morphological flattening, and decreased proliferation. Further, in these cells, PVT1 knockdown leads to reduction in c-MYC protein levels [29]. These observations raise the possibility that retention of c-MYC mediated by the large hyperacetylated domains blocks NMC cell differentiation.

### 6.2. BRD2 and BRD4 Interact with Viral Proteins and Contribute to Oncogenesis in Host Cells Infected with Viruses

Kaposi’s sarcoma-associated herpesvirus (KSHV) causes Kaposi’s sarcoma (KS), a cancer commonly occurring in AIDS patients, as well as primary effusion lymphoma, and some types of multicentric Castleman’s disease. Its latent nuclear antigen (LANA), which is expressed in the nuclei of latently infected cells, mediates episomal replication and persistence of viral genomes [111]. Yeast two-hybrid screening using a carboxy-terminal fragment of LANA fused to the DNA binding domain of GAL4 (bait) and a human leukocyte cDNA library expressing proteins fused to the GAL4 activation domain (prey) identified the BRD2 protein as an activator of reporter gene expression, suggesting that LANA interacts with BRD2 [112]. Further, a pull-down assay of lysates from BCP-1 cells, a KSHV-infected B-cell lymphoma cell line, with an anti-BRD2 antibody results in the immunoprecipitation of both BRD2 and LANA, indicating interaction between BRD2 and LANA in vivo [112]. An immunoprecipitation assay using lysates from HEK 293T cells co-transfected with a series of LANA deletion constructs and a GFP-tagged BRD2 construct shows that deletions within the carboxy terminus prevent LANA from co-immunoprecipitating with an anti-GFP antibody, suggesting that the carboxy-terminal domain of LANA interacts with BRD2 [113]. Pull-down assays of lysates from SF9 insect cells expressing the recombinant LANA protein using glutathione beads coated with a series of GST-tagged BRD2 deletion proteins show that deletions within the ET domain interfere with the binding of BRD2 to LANA, suggesting that the ET domain of BRD2 interacts with LANA [112]. These results indicate that the carboxy-terminal domain of LANA interacts with a region in BRD2 that contains the ET domain. Like the interaction of LANA with BRD2, BRD4 short has been reported to interact directly with an element in the LANA carboxy-terminal domain through its carboxy-terminal region that contains the highly conserved ET domain [114,115]. Immunohistochemical staining of KSHV-infected BCLM cells shows clear colocalization of BRD4 with LANA in punctate speckles, wherein KSHV episomes are colocalized [111] on both mitotic chromosomes and interphase nuclei [114].

Papillomaviruses constitute a group of small DNA viruses that cause benign tumors in a variety of higher vertebrates. During the latent infection period, viral genomes persist as episomes in the nuclei of proliferating host cells. The papillomavirus genome includes an early region (E) encoding six (E1, E2, E4, E5, E6, and E7) open reading frames (ORF) that are expressed immediately after initial infection of the host cell. The E2 protein, which binds to the viral replication origin cooperatively with the E1 protein, plays a crucial role in long-term episomal maintenance and DNA replication of the viral genome [116,117]. The E6 protein mediates p53 degradation resulting in reduced ability to respond to DNA damage in host cells [118]. Because the E2 regulatory protein suppresses the enhancer and promoter activity that causes E6 and E7 transcription, integration of the papillomavirus DNA into cellular chromosomes in a manner that disrupts the E2 ORF, promotes oncogenesis in host cells [119,120]. The viral proteins need to interact with a variety of cellular proteins to efficiently execute their function.

Cellular E2-interacting proteins have been explored using the bovine papillomavirus (BPV) E2 protein. A proteomic analysis of nuclear lysates from human cervical carcinoma C33 cells transduced with a recombinant retrovirus expressing FLAG-HA-tagged BPV E2 shows that BRD4 is co-immunopurified with anti-FLAG and anti-HA antibodies, suggesting that BRD4 is a member of the cellular interacting partners of BPV E2 [121]. The overlap of E2 and BRD4 immunofluorescence images in E2-transfected C33 cells demonstrates that these two proteins colocalize on mitotic chromosomes [121]. In vitro binding assays of fragments from BRD4 covering different regions of the protein with the GST-E2 fusion protein immobilized on glutathione resin, show that deletion of the C-terminal region prevents BRD4 from binding to GST-E2, indicating that E2 binds to the BRD4 C-terminal domain [121]. Further, an immunohistochemical study of E2-transfected C33 cells stably expressing the BRD4 C-terminal domain shows disappearance of overlapping E2 and BRD4 staining, suggesting that expression of the BRD4 C-terminal domain blocks E2 interaction with BRD4 in a dominant-negative manner [121]. Collectively, these findings indicate that interaction of the BPV E2 protein with BRD4 is required to tether the viral genome to the host mitotic chromosomes.

The E2 protein encoded by human papillomaviruses (HPVs) has also been reported to be associated with BRD4. Using nuclear extracts of human embryonic kidney-derived 293 cells that express FLAG-tagged HPV E2, anti-FLAG immunoaffinity purification followed by mass spectrometry identified BRD4 as a cellular protein that is naturally associated with HPV E2 [122]. In vitro reconstituted chromatin transcription experiments illustrate that BRD4 confers the ability to silence HPV chromatin transcription on E2. BRD4 knockdown in C33 cells using an shRNA expressing retroviral vector causes a reduction in E2-mediated repression of E6 promoter activity, suggesting that BRD4 is a cellular co-repressor essential for E2-mediated repression of HPV E6 promoter activity [122]. Chromatin samples isolated from E2-expressing HeLa cells that harbor endogenous HPV genomes, using anti-TAF1 and Pol II antibodies, do not include DNA from the E6 promoter region, suggesting that E2 complexed with BRD4 blocks the recruitment of TAF1 (a subunit of TFIID) and RNA Pol II to the HPV E6 promoter region [122]. Further, a genome-wide siRNA screen using BPV E2-expressing C33 cells transfected with an E2-repressible reporter gene identified BRD4 as a cooperator contributing to E2-mediated transcriptional repression [123]. In contrast, both competitive inhibition of E2 binding to BRD4 by the C-terminal fragment of BRD4 and BRD4 knockdown by siRNA compromise transcriptional activation from an E2-responsive promoter, suggesting that BRD4 is required to mediate E2 transcriptional activation function [124,125]. Further, transfection of E2 fused with Cdk9, a subunit of P-TEFb, in C33 cells more efficiently enhances the activity of E2-responsive reporter genes, indicating that the recruitment of P-TEFb by BRD4 is important for E2-dependent transactivation [126]. Notably, BRD4 has dual effects on the transcriptional regulation of E2 in papillomaviruses.

### 6.3. BET Proteins Are Potential Therapeutic Targets in a Wide Range of Cancers

To identify and optimize therapeutic lead compounds for translation of small-molecule modulators of epigenetic targets as cancer therapeutics, high-throughput screening has been conducted. JQ1, a thienotriazolodiazepine, is identified as a potent and selective small-molecule inhibitor of BET proteins that competitively binds to the acetyl-lysine recognition pocket of their bromodomains [30]. FRAP assays with human osteosarcoma cells transfected with GFP-BRD4 and GFP-BRD4-NUT reveal accelerated fluorescence recovery in the presence of JQ1, indicating that JQ1 displaces BRD4 from nuclear chromatin and increases the levels of freely diffusing BRD4 [30]. Treatment of the NMC patient-derived cell line with JQ1 eliminates discrete nuclear foci composed of the BRD4-NUT oncoprotein, arrests proliferation at the G1 cell-cycle stage, and prompts terminal squamous differentiation. Analysis of NMC xenografts in mice by positron-emission tomography (PET) imaging of ^18^F-fluorodeoxyglucose (FDG) uptake shows that JQ1-treated mice exhibit marked reduction of FDG uptake, whereas vehicle-treated mice develop progressive cancer. In fact, remarkable tumor regression and prolonged survival are observed in mice treated with JQ1 compared to that in the vehicle control [30]. These observations highlight the immediate therapeutic potential of direct-acting inhibitors of BET proteins.

c-MYC is a transcription factor that regulates cell proliferation. Aberrant c-MYC expression leads to cancer by the coordinated activation of transcriptional pathways involved in cell division, metabolic adaptation, and survival. c-MYC is thus viewed as a promising target for anti-cancer drugs. To investigate whether BET inhibition specifically abrogates MYC-dependent transcription, global transcriptional profiling and unbiased gene set enrichment analysis (GSEA) [127] was performed with human multiple myeloma (MM) cell lines that highly express BET proteins. The result showed that downregulation by JQ1 is strongly correlated with canonical transcriptional signatures of MYC-dependent genes, indicating that BET bromodomain inhibition by JQ1 gives rise to selective repression of the transcriptional network induced by c-MYC [31]. JQ1 treatment in tumor-bearing SCID mice orthotopically xenografted with an intravenous injection of MM cells significantly decreases the disease burden and results in marked prolongation of overall survival compared to that in vehicle-treated animals [31]. Likewise, small-molecule BET inhibitors (JQ1 and I-BET) show anticancer effects in vitro and in vivo on acute myeloid leukemia (AML) [32,33], mixed lineage leukemia (MLL) [128], Burkitt’s lymphoma [33], medulloblastoma, which is the most common brain tumor in childhood [129], *MYCN*-amplified neuroblastoma [130], castration-resistant prostate cancer [131], Ewing’s sarcoma [132], and colorectal cancer [133]. These results demonstrate the pre-clinical efficacy of BET inhibitors in a wide range of malignancies, which reinforces the therapeutic potential of these drugs. Currently, clinical trials are under way by means of small-molecule bromodomain inhibitors to treat some of the most common cancers, including lymphomas, leukemias, multiple myelomas and solid tumours (such as cancers of the pancreas and prostate) [134].

To optimize the clinical efficacy of BET inhibition, mechanisms of drug resistance have been evaluated. Comparison between I-BET-resistant MLL cells and vehicle MLL cells by GSEA reveals the enrichment of leukemia stem cell signatures and significant upregulation of Wnt/β-catenin signaling in the resistant clones. Negative regulation of the Wnt/β-catenin pathway results in restoration of sensitivity to I-BET both in vitro and in vivo [135]. A chromatin-focused RNAi screen in mouse AML cells shows that suppression of PRC2 (Polycomb Repressive Complex 2) histone methyltransferase to transcriptionally silence chromatin, promotes JQ1 resistance in AML. GSEA with the transcriptomes of JQ1-resistant AML cells generated by PRC2 shRNA and JQ1-sensitive AML cells reveals that loss of PRC2 can facilitate transcriptional activation of Wnt signaling, which can drive *MYC* transcription [136]. These results identify and validate Wnt signaling as a driver and candidate biomarker of BET resistance in leukemia. Genome-wide shRNA “dropout screens” for functional genomic analyses on 77 breast cancer cell lines show that BRD4 is preferentially essential for cell viability in luminal/HER2 lines. Although many luminal/HER2 lines that are sensitive to BRD4 knockdown are JQ1-resistant, most basal lines sensitive to BRD4 knockdown are JQ1-sensitive. Further, integrative analysis reveals a strong correlation between JQ1 resistance and *PIK3CA* (phosphatidylinositol-4,5-bisphosphate 3-kinase catalytic subunit alpha) mutation, which is supported by the fact that PIK3CA overexpression confers JQ1 resistance to JQ1-sensitive cells whereas a PIK3CA-specific inhibitor increases JQ1 sensitivity in resistant cells [137]. These findings highlight the identity of BRD4 as a potential therapeutic target and the significance of *PIK3CA* mutation leading to BET-inhibitor resistance in luminal breast cancer.

### 6.4. BRDT Is a Potential Target for Male Contraception

An ITC study, which has been performed to confirm the binding of a synthetic H4Kac4 peptide to human BRDT bromodomains in the presence and absence of JQ1, shows that the peptide binds to modules with sufficiently low *K_d_* values and this protein-protein interaction is directly inhibited by JQ1 [138]. Intraperitoneal injection of JQ1 into male mice for several weeks reduces the seminiferous tubule area, testis size, and spermatozoa number and motility without affecting the hormone levels [138]. To define the consequences of BRDT inhibition by JQ1, genome-wide expression analysis and GSEA have been carried out with testes RNA from vehicle- and JQ1-treated mice. The result showed that genes downregulated by JQ1 are enriched with functionally defined gene sets reflecting pachytene spermatocyte and spermatid transcriptional signatures, suggesting that JQ1 inhibition selectively depletes germ cell transcripts. Although male mice treated with an appropriate dose of JQ1 for a suitable period mate normally and are completely infertile, fertility is restored after JQ1 treatment is stopped [138]. These findings provide evidence that small-molecule inhibitors of BRDT are potential lead compounds for male contraception.

## 7. Concluding Remarks

This review focuses on the basic functions of the four paralogous BET proteins, which are summarized in Table 1 together with the pathological functions of the proteins. These proteins have a common feature of epigenetic transcriptional regulators that recognize acetylated lysines in histones through their bromodomains and control the movement of RNA Pol II on chromatin. The four paralogous BET proteins are structurally classified into a group including BRD2 and BRD3 that does not possess the CTD and a group of BRD4 and BRDT that possesses the CTD. However, some splice variants of BRD4 and BRDT do not have the CTD. BRD2 and BRD3 facilitate RNA Pol II elongation in transcription through their histone chaperone activities that are attributed to the interaction between bromodomains and acetylated chromatin. BRD4 also has a histone chaperone activity. In addition, BRD4 enhances the recruitment of P-TEFb by interacting with the CTD, which causes Ser2 phosphorylation of the RNA Pol II CTM and leads to release of Pol II from a pause in transcription elongation in the promoter-proximal region. Further, BRD4 is associated with distal enhancers that play critical roles in developmental and transcriptional programs. BRDT also recruits P-TEFb interacting with its CTD; however, detailed roles of BRDT in transcription remain to be elucidated. BRDT is a key molecule participating in chromatin remodeling by interacting with hyperacetylated nucleosomes through its bromodomains and plays a pivotal role in the replacement of acetylated histones at the post-meioitic stages during spermatogenesis. To fully elucidate the exact processes of transcriptional regulation by BET family proteins, it is worthwhile to investigate how BET proteins “read and translate” histone modifications in cellular proliferation and differentiation during development and spermatogenesis.

Secondly, the review focuses on the pathological functions of BET family proteins. BRD2 and BRD4-S (a BRD4 short variant) interact with the LANA of KSHV, which is required for episomal replication and persistence of viral genomes, through their ET domain. The association of the HPV E2 protein with BRD4 is required to tether the viral genome to host mitotic chromosomes, and BRD4 is essential for E2-mediated transcriptional regulation. The BRD4-NUT fusion protein, which is produced by a gene reconstructed by a chromosomal translocation, activates multiplication related genes by hyperacetylating their chromatin with recruited p300, which causes NUT midline carcinoma (NMC). Treatment of NMC-patient derived cells with JQ1, a potent and selective small-molecule inhibitor of BET proteins, arrests proliferation at the G1 cell-cycle stage and prompts terminal squamous differentiation. Global transcriptional profiling of human multiple myeloma cells treated with JQ1 reveals a strong correlation between the genes downregulated by JQ1 and the genes comprising MYC-dependent transcriptional pathways. Small-molecule BET inhibitors including JQ1 have immediate therapeutic potential for a wide range of malignancies. Although BET inhibitors have a dramatic effect on suppression of tumor growth, there is a possibility that they cause critical damage to normal cell proliferation that is controlled by the MYC-dependent transcriptional pathway and the Wnt signaling pathway. Furthermore, as revealed by the administration of JQ1 to mice, BET inhibitors might have deleterious effects on the transcriptional regulation underlying human learning and memory. Further basic studies should be required to investigate various effects of BET inhibitors. Of many derivatives of BET inhibitors, compounds without harmful effects should be strictly selected. For example, a compound that cannot cross the blood-brain barrier would be available as an anti-cancer drug. To further develop BET inhibitors as anti-cancer drugs towards clinical application, the biological pathway leading BET inhibitor resistance needs to be investigated through a detailed molecular study.

## Figures and Tables

**Table 1:** Functions of mammalian Bromodomain and Extra-Terminal Domain (BET) proteins. LANA, latent nuclear antigen; KSHV, Kaposi’s sarcoma-associated herpesvirus; INF, interferon; BPV, bovine papillomavirus; HPVs, human papillomaviruses.

BET Protein	Functions	References
BRD2	• Promotion of E2F-dependent cell cycle progression in HeLa and HEK293 cells	[47,48]
	• Closure of the neural tube in mouse embryos	[49,50]
	• Maintenance of the number of GABAergic neurons in the neocortex and the striatum of mice	[52]
	• Assist of transcription in hyperacetylated chromatin (Property of histone-chaperone)	[23]
	• Transcriptional activation of *HOXA11*and *D11* in HEK293 cells	[26]
	• Enhancement of GATA1-mediated erythroid gene activation	[63]
	• Interaction with LANA of KSHV that mediates episomal replication and persistence of viral genomes	[112,113]
BRD3	• Assist of transcription in hyperacetylated chromatin (Property of histone-chaperone)	[23]
	• Transcriptional activation of *HOXB3*, *B4*, *B5*, *B6*, *C8*, *C9*, *C10*, *A3*, *A5*, *A6*, and *A7* in HEK293 cells	[26]
	• Enhancement of GATA1-mediated erythroid gene activation	[63]
	• Carcinogenesis induced by BRD3-NUT fusion protein	[106]
BRD4	• Stimulation of G2/M transition in HeLa cells	[8]
	• Cell cycle progression in P19 embryonic carcinoma cells	[19]
	• Maintenance of inner cell mass in mouse blastocysts	[66]
	• Transcriptional activation of Nanog required for maintaining the pluripotency of ES cells	[67]
	• Release from a pause in transcription elongation	[21,24]
	• Assist of transcription in hyperacetylated chromatin (Property of histone-chaperone)	[25]
	• Transcriptional activation of *c-Myc* and *Klf4* in NIH3T3 cells	[25]
	• Transcriptional activation of *HOXB2*, *B3*, *B4*, *B5*, *B6*, *B7*, *B8*, *A4*, and *C5* in HEK293 cells	[26]
	• Transcriptional regulation of genes involved in learning and memory in mice	[34]
	• Enhancement of INF-induced gene transcription	[77]
	• Signal transducer of the cellular response to oxidative stress	[82]
	• Prevention of splicing inhibition in heat stress-induced cells	[83]
	• A gene bookmark for transcriptional reactivation in post-mitotic cells	[87,89]
	• Carcinogenesis induced by BRD4-NUT fusion protein	[28,106]
	• Interaction with LANA of KSHV that mediates episomal replication and persistence of viral genomes	[114,115]
	• Tethering of BPV genome to host mitotic chromosomes	[121]
	• Transcriptional regulation of E2 that mediates episomal maintenance and DNA replication of HPV genome	[122,124,125]
BRDT	• Transcriptional regulation of genes responsible for meiotic progression during spermatogenesis	[27]
	• Splicing machinery in testicular cells	[96]
	• Chromatin remodeling in MEL, 3T3, and COS7 cells	[95,98,101]
	• Histone replacement at post-meiotic stages during spermatogenesis	[27]
